# Arcuate Nucleus Orexin-A Signaling Alleviates Cisplatin-Induced Nausea and Vomiting Through the Paraventricular Nucleus of the Hypothalamus in Rats

**DOI:** 10.3389/fphys.2018.01811

**Published:** 2018-12-17

**Authors:** Feifei Guo, Shengli Gao, Luo Xu, Xiangrong Sun, Nana Zhang, Yanling Gong, Xiao Luan

**Affiliations:** ^1^Pathophysiology Department, School of Basic Medicine, Qingdao University, Qingdao, China; ^2^Department of Clinical Laboratory, The Affiliated Hospital of Qingdao University, Qingdao, China; ^3^Department of Pharmacy, College of Chemical Engineering, Qingdao University of Science and Technology, Qingdao, China

**Keywords:** arcuate nucleus, orexin-A, cisplatin-induced nausea and vomiting, paraventricular nucleus, rats

## Abstract

The most common side effects of cisplatin chemotherapy are nausea and vomiting, and the overwhelming majority of research studies on the mechanism of cisplatin-induced nausea have been focused on the “vomiting center.” As a modulatory center of gastric motility, the roles of the hypothalamus in nausea and vomiting remain unclear. In the present study, we investigated the effects of exogenous orexin-A injected into the arcuate nucleus (ARC) on cisplatin-induced nausea and vomiting, and the possible underlying mechanism. Kaolin intake was calculated daily in cisplatin-treated and saline-treated rats. Gastric motility recording, injections into the ARC, and lesions of the paraventricular nucleus (PVN) were used to study the effects of orexin-A and the hypothalamic nucleus on disorders of gastrointestinal function in cisplatin-treated rats. The pathway from the ARC to the PVN was observed through Fluoro-Gold retrograde tracing. Furthermore, an NPY Y1 receptor antagonist was administered to explore the possible mechanisms involved in the effects of orexin-A in the ARC. We illustrated that exogenous orexin-A injected into the ARC reduced kaolin intake and promoted gastric motility in cisplatin-treated rats, and these effects could have been blocked by an ipsilateral PVN lesion or co-injected antagonist of orexin-A-SB334867. Additional results showed that orexin-A-activated neurons in the ARC communicated directly with other neurons in the PVN that express neuropeptide Y (NPY). Furthermore, activation of the downstream NPY pathway was required for the observed effects of orexin in the ARC on cisplatin-induced nausea and vomiting. These findings reveal a novel neurobiological circuit from the ARC to the PVN that might provide a potential target for the prevention and treatment of cisplatin-induced nausea and vomiting.

## Introduction

Cisplatin, as a chemotherapeutic agent, plays a core role in the multidrug chemotherapy of various malignancies, such as head and neck cancer, lung cancer and cervical cancer, etc. (Chen et al., 2017; Steuer et al., 2017; Szturz et al., 2018). However, it is also associated with a variety of side effects, such as nephrotoxicity, myelosuppression, and some gastrointestinal disorders like nausea, vomiting, and anorexia (Janicki, 2016). These symptoms adversely and severely affect the quality of life, reduce resistance to disease, and prevent completion of the course of chemotherapy. Serotonin (5-HT) receptor antagonist and neurokinin-1 receptor antagonists have been used to prevent nausea and vomiting caused by chemotherapy. However, many patients still experience severe gastrointestinal dysfunction, particularly in the later stages of treatment (Lorusso, 2016).

The regulation of feeding and gastrointestinal motility involves a complex integration of central and peripheral signals in the brain. During the process, appetite-related neuropeptides are the major neurotransmitters in the central nervous system, and they have been found to regulate cisplatin-induced reductions in food intake (Yakabi et al., 2010; Yamamoto et al., 2014). In cisplatin-treated rats, previous studies reported that most appetite-related peptides were modulated to exert anorexigenic effects, such as an increase in the levels of two key anorexigenic peptides, proopiomelanocortin (POMC) and the cocaine- and amphetamine-regulated transcript (CART), as well as the inhibition of the orexigenic peptides, neuropeptide Y and ghrelin (Liu et al., 2006; Yoshimura et al., 2013). Intraperitoneal injection of ghrelin greatly alleviated the increased gastric contents and the reduced feeding caused by cisplatin in rats and mice (Liu et al., 2006). Furthermore, a previous study reported that administration of exogenous ghrelin stimulated food intake and minimized adverse events in patients with esophageal cancer who also received cisplatin-based neoadjuvant chemotherapy (Hiura et al., 2012). However, the orexigenic peptide, orexin was elevated in the hypothalamus by cisplatin (Yoshimura et al., 2013), as a counterregulation to anorexia. The reasons behind the increased levels of orexin and its functions following cisplatin administration remain unknown. Similarly, whether orexin, the peptide increased by cisplatin, affects food intake and nausea in cisplatin-treated rats remains unknown.

Orexin is an important feeding-related peptide that plays a role in arousal (Piper et al., 2000), reward-seeking behavior (Aston-Jones et al., 2010), energy homeostasis, sensory modulation, stress processing, endocrine functions, etc. (Boss, 2014; Christopher, 2014). Orexin has two active forms, orexin-A and orexin-B, both of which are cleaved from the common precursor prepro-orexin (Gatfield et al., 2010). Orexin-A is a 33 amino-acid peptide that activates both orexin-1 and orexin-2 receptors with similar potencies; whereas orexin-B, a 28 amino-acid peptide, is mainly selective for the activation of orexin-2 receptors (Matsuki and Sakurai, 2008). Orexin is believed to improve gastric acid secretion and gastrointestinal motility, and increase food intake and body weight (Baccari, 2010; Bulbul et al., 2010). Orexin-containing neurons in the lateral hypothalamic area (LHA) activate other neurons in the dorsal motor nucleus of the medulla oblongata. This, in turn, stimulates gastric acid secretion, gastric motility, and pancreatic secretion, and induces gastroprotective effects. These findings suggest that reduced orexin signaling might play a role in the pathogenesis of functional gastrointestinal disorders (Okumura and Nozu, 2011).

The arcuate nucleus (ARC) is an aggregation of neurons, adjacent to the third ventricle and the median eminence that expresses an abundance of orexin receptor 1. The neurons in the ARC are responsible for receiving sensory signals, integrating information, and providing inputs to other nuclei in the brain. The orexin-A excited GABAergic, leptin-responsive and NPY-containing neurons in the ARC, and it is involved in the whole-body O_2_ consumption (Rauch et al., 2000; Yamanaka et al., 2000; Burdakov et al., 2003; Wang et al., 2003). However, the role of orexin in the ARC of cisplatin-treated rats still needs to be determined. Moreover, it is unclear whether the signals induced by orexin in the ARC are transmitted to another hypothalamic nucleus, or whether the effects of orexin in the ARC depend on other neuropeptides. In the present study, we selected one of the most important hypothalamic feeding nuclei (PVN) as the target to determine a novel orexigenic pathway.

To observe the role of orexin in cisplatin-induced emesis, the changes in kaolin intake before and after orexin-A injection into the ARC, the effect of PVN lesions on the function of the orexin injection, and the connection between ARC OX1R neurons and PVN NPY neurons were studied. Furthermore, the NPY Y1 receptor antagonist, BB3304 was administered to explore the mechanism of orexin in the ARC. This study focused on the promoting effects of orexin on the gastric motility in ARC-PVN pathways. It is a potential novel way or strategy to alleviate the nausea and vomiting induced by cisplatin chemotherapy and improve the life quality of patients suffered from cancer. However, there is still a long way to explore the safety and efficacy of orexin before it is used in clinics.

## Materials and Methods

### Animals

Wistar rats (Male, 250∼300 g, Qingdao Institute for Drug Control, Shandong, China) were housed under controlled conditions of temperature (25 ± 2°C) and light (08:00 a.m. to 08:00 p.m.), with *ad libitum* access to food and water. Protocols (number: 0013219) were approved by the Qingdao University Animal Care and Use Committee.

### Implantation of Brain Cannula

Rats had been fasted overnight and anesthetized with Inactin (100 mg/kg, Sigma-Aldrich Chemical, United States) and placed in a stereotaxic frame. A 24-gauge stainless steel guide cannula was implanted into the ARC (bregma: P: -2.1∼-4.3 mm, L (R): 0.2∼0.5 mm, H: 9.8∼10.3 mm) or the lateral ventricle (bregma: P: -2.5 mm, L (R): 1.3 mm, H: 7.7 mm), in accordance with the guidelines of the atlas of Paxinos and Charles (2013). After a one-week recovery period, the drugs were administered with an injection cannula (29-gauge) connected to a syringe by a 10-cm piece of polyethylene tubing.

### Kaolin Intake

Kaolin pellets were prepared according to a previously reported method (Yamamoto et al., 2011). Briefly, pharmaceutical-grade kaolin (hydrated aluminum silicate) was mixed with 3% (w/w) gum arabic in distilled water, to form pellets similar in size to chow pellets. These pellets were then completely dried at room temperature (25 ± 2°C).

Daily kaolin consumption was measured, following Kouichi’s method (Yamamoto et al., 2014). Rats were allowed to adapt to the experimental cages for 1 week, and given free access to water, food pellets, and kaolin throughout the experimental period. The weight of kaolin consumed was monitored daily, and the data were stored and analyzed on a computer.

### Gastric Motility

Gastric motility measurements were conducted according to our previously described methods (Guo et al., 2015). Rats were fasted overnight and anesthetized with Inactin (100 mg/kg, i.p.; Sigma-Aldrich Chemical, United States). Their stomachs were subsequently exposed via laparotomy. At the serosa of the gastric antrum, 0.5 cm caudal to the pyloric ring, a strain gauge was sutured to measure muscle contractions. The strain gauge sent muscle signals via the lead wire that extended subcutaneously, and was terminally fixed at the nape of the neck with approximately 2–3 cm outside. After recovery for 3 days, the gastric motility of rats fasted overnight was recorded on a polygraph (3066–23; Chengdu Precision Instruments, Sichuan, China), by connecting the lead wires.

### PVN Electric Lesion

The method of inflicting the electrical lesion was similar to that employed in a previous study (Dube et al., 2006). Briefly, rats were anesthetized with Inactin and the coordinates of the PVN were measured with a stereotaxic instrument, in accordance with the atlas of Paxinos and Watson (Paxinos and Charles, 2013): 1.8 mm caudal to bregma, 0.4 mm lateral to the midline, and 7.9 mm ventral to the dorsal surface. After craniotomy, an electrode with an exposed tip of 0.5 mm was inserted into the PVN. Anodal direct current of 1 mA for 20 s was used to destroy the nucleus. For the sham lesion, the same procedure was employed; however, no current was allowed to pass through the electrode. The opening in the skull was then sealed with dental cement and the rats were allowed to recover for 3 days.

The extent of the unilateral electrical PVN-targeted lesion was determined postmortem by immunohistochemical detection of the neuronal marker NeuN (rabbit, 1:500, Abcam, Cambridge, United Kingdom, cat# ab104225). The rats with minimal or misplaced lesions were excluded from the analysis.

### Fluoro-Gold Retrograde Tracing

After anesthetization, rats were mounted on a stereotaxic apparatus. Retrograde tracing from the PVN followed unilateral pressure injection of Fluoro-Gold (FG, Sigma-Aldrich Chemical, MO, United States; 2% in 0.9% NaCl). Pressure injections were performed using a microinfusion pump with a volume of 200 nL. After a 7-day recovery, rats were perfusion-fixed with 4% paraformaldehyde. The brains were removed for post-fixing, dehydrating, embedding, and sectioning. Photographs of the fluorophores were taken under a Leica DM6000B fluorescence microscope (Leica Microsystems AG, Wetzler, Germany).

### Immunohistochemistry

The anesthetized rats were perfused transcardially with ice-cold normal saline, followed by 4% paraformaldehyde phosphate buffer solution. The brains were routinely removed for embedding, freezing, and sectioning. The primary antibody incubation was conducted overnight at 4°C. After washing, the sections were incubated with secondary antibody for 2 h at room temperature. After washing off the unbound secondary antibodies, the sections were mounted in Citifluor (Citifluor, London, United Kingdom). The photographs of fluorophores were taken under a Leica DM6000B fluorescence microscope (Leica Microsystems AG, Wetzler, Germany).

### Effect of Orexin-A on Kaolin Intake in Rats

Sixty rats were randomly divided into six groups: (1) saline + saline (SS); (2) cisplatin + saline (CS); (3) cisplatin + orexin-A (CO); (4) saline + SB-334867 (SSB); (5) cisplatin + SB-334867 (CSB); and (6) cisplatin + orexin-A + SB-334867 (COSB) groups. Unilateral cisplatin, orexin-A, and SB-334867 dosages were determined based on preliminary experiments and previous research (Yoshimura et al., 2013; Hao et al., 2016). In the SS and SSB groups, a single intraperitoneal injection of saline was first administered, and then 30 min later an injection into the ARC of saline or SB-334867 (orexin-1 receptor antagonists, 5.0 μg) was administered once daily. A single injection of cisplatin (6 mg/kg, IP) was administered to rats in the CS, CO, CSB, and COSB groups. A single daily injection of saline was administered to the ARC of rats in the CS group; similarly, a single daily injection of orexin-A (0.5 μg) was administered to rats in the CO group; SB-334867, administered to the CSB group; and a mixture of orexin-A and SB-334867 administered to the COSB group.

The daily kaolin consumption was measured on the day before and days 1, 3, and 5 after the cisplatin injection. Rats were administered the respective drugs at 18:00 h, and their daily kaolin consumption was measured.

### Effect of Orexin-A Injection on Gastric Motility

Sixty rats were randomly divided into six groups, similar to experiment 1. On day 0, cisplatin was administered to rats that were subsequently evaluated on the third day. Gastric motility was recorded for 1 h separately, before and after administration of the drugs. The changes in gastric motility were evaluated according to the percentage motor index (% MI) of motor activity by the following formula: (area under the manometric trace for the 30-min period after drug administration)/(area under the manometric trace for the 30-min period before drug administration) × 100%.

### Determination of Whether Unilateral PVN Lesions Block the Effects of Activation of the Ipsilateral ARC OX-1R-Expressing Neurons

Rats were subjected to a unilateral electrical lesion targeted at the PVN (*n* = 10) or sham lesions (*n* = 7). Before the night cycle (lights off at 8 p.m.), 0.5 μg orexin-A was administered to the ARC, ipsilateral to the PVN lesion. Kaolin intake was recorded at 12 h after administration (kaolin spillage accounted for). Treatments were separated by an intervening day using a counterbalanced mixed design, with sham or PVN lesions as a between-subjects variable, and the drug as the within-subjects variable.

In a separate experiment with the same cohort, 0.5 μg orexin-A was administered to the ARC, contralateral to the PVN lesion.

### Investigation of PVN Input From ARC OX1R-Expressing Neurons

To investigate the PVN input from the ARC, FG retrograde tracing and immunohistochemistry were used. Each rat (*n* = 8) received a unilateral injection of FG targeted at the PVN. The rats were fixed by 4% paraformaldehyde perfusion 7 days later. Their brains were removed and dealed with a way as described in *FG Retrograde Tracing*. FG was examined using its native fluorescence. Immunohistochemistry followed the steps described above, and the following antibodies were used: Anti-OX1R primary antibody (rabbit, 1:500, Abcam, Cambridge, United Kingdom, cat# ab68718); anti-rabbit Alexa Fluor 594 secondary antibody (goat polyclonal, 1:500, Jackson ImmunoResearch, PA, United States, cat# 112-585-003).

Quantification of colocalization of FG and OX1R positive cells was done with ImageJ software by an experimenter who was blind to the treatment conditions. The calculation was based on the number of colocalized FG and OX1R cells divided by the total number of FG positive cells.

### Effects of ARC Orexin-A Injection on c-Fos Expression in PVN Neurons

Rats were subjected to unilateral ARC orexin-A (*n* = 8; 0.5 μg) or vehicle injections (*n* = 5). Ninety minutes later, rats were euthanized and their brains were removed and processed, according to the procedures outlined in *Immunohistochemistry*.

C-Fos is regarded as a marker of neuronal activity, and immunohistochemical detection was performed following the procedure described in *Immunohistochemistry*, using a mouse monoclonal anti-c-Fos primary antibody (1:100, Santa Cruz Biotechnology, CA, United States, cat# sc-8047), followed by a goat anti-mouse secondary antibody conjugated to Cy3 (1:500, Jackson ImmunoResearch, PA, United States, cat# 115-165-003). Immunohistochemistry detection of NPY was performed using a rabbit anti-NPY polyclonal antibody (1:500, Abcam, Cambridge, United Kingdom, cat# ab30914), and was followed by incubation of a goat anti-rabbit secondary antibody (FITC, 1:1000, Abcam, Cambridge, United Kingdom, cat# ab6717).

Quantification of colocalization of c-Fos and NPY positive cells was processed according to the means described above. This calculation was based on the number of co-localized c-Fos and NPY cells divided by the total number of NPY positive cells.

### Effect of Intracerebroventricular Injection of an NPY Receptor Antagonist on the Role of ARC Orexin-A in Kaolin Intake of Cisplatin-Treated Rats

Twenty rats were subjected to cannula implantation that targeted both the ARC and lateral ventricle. Rats were randomly divided into two groups: (1) the cisplatin + saline + orexin-A (CSO) group and (2) the cisplatin + BB3304 + orexin-A (CBO) group. After cisplatin treatment, rats were subjected to lateral ventricular injections of 60 μg of the NPY-Y1 receptor specific antagonist, BB3304, immediately followed by 0.5 μg orexin-A to the ARC. The dose of BB3304 was determined based on previous literature (Yamanaka et al., 2000). Treatments were separated by an intervening day, using a counterbalanced within-subjects design. Kaolin intake was performed on the days 1, 3, and 5 after cisplatin administration (kaolin spillage accounted for).

### Statistics

The analyses were carried out using the SPSS software (version 17, SPSS Inc., Chicago, IL, United States) and results are presented as mean ± SD. All statistical analyses employed repeated measures or one-way ANOVA. *P* < 0.05 was considered statistically significant.

## Results

### Effect of Orexin-A on Kaolin Consumption in Cisplatin-Treated Rats

**FIGURE 1:**
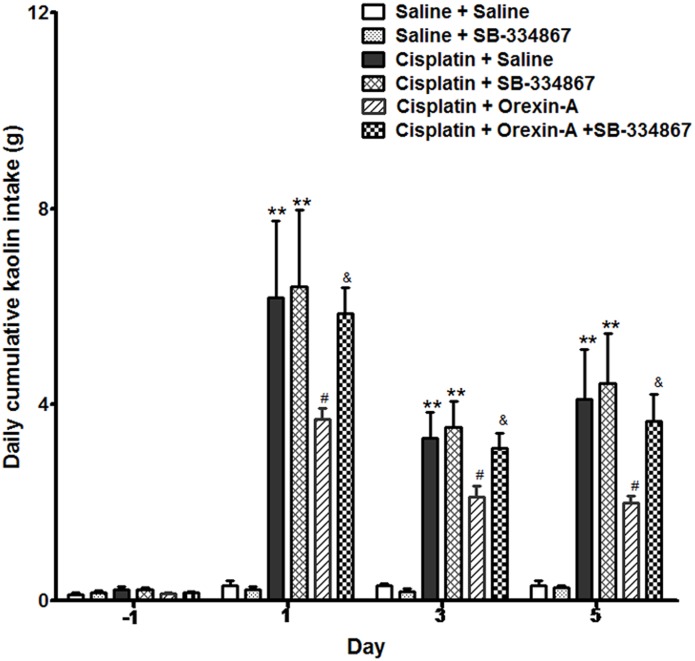
Effect of orexin-A injection into the ARC on kaolin consumption in cisplatin-treated rats. Kaolin intake of the cisplatin + saline group was significantly higher than that of the saline + saline group. The orexin-A injection into the ARC inhibited kaolin intake induced by cisplatin in the cisplatin + orexin-A group, that was partly blocked by the orexin receptor 1 antagonist SB-334867 in the cisplatin + orexin-A + SB-334867 group. The SB-334867 alone did not affect kaolin intake in the saline + SB-334867 and cisplatin + SB-334867 groups. ^∗∗^*P* < 0.01 vs. SS group; ^#^*P* < 0.05 vs. CS group; ^&^*P* < 0.05 vs. CSB group, *n* = 10. ARC, arcuate nucleus; CS, cisplatin + saline; CSB, cisplatin + SB-334867; SS, saline + saline.

Rats ingested a small amount of kaolin (range 0–0.3 g) during the habituation period and observation periods following the intraperitoneal administration of saline. The daily cumulative kaolin consumption of days 1, 3, and 5 after cisplatin treatment were 6.25 ± 1.59, 3.29 ± 0.74, and 4.38 ± 1.41 g (*P* < 0.01, vs. pretreatment). In the CO group, orexin-A injected into the ARC reduced the kaolin intake daily to 3.59 ± 0.32, 2.09 ± 0.22, and 1.76 ± 0.19 g (Figure 1), values that were significantly lower than those of the CS group (*P* < 0.05). The effect of orexin on kaolin consumption was inhibited by the orexin-1 receptor antagonist SB-334867 (*P* < 0.05, vs. CO group). Administration of SB-334867 alone did not affect kaolin consumption (*P* > 0.05, SSB vs. SS and CS vs. CSB groups, Figure 1). The results indicated that daily administration of orexin-A in the ARC effectively inhibited kaolin intake in cisplatin-treated rats.

### Effect of Orexin-A on Gastric Motility in Rats Treated With Cisplatin

**FIGURE 2:**
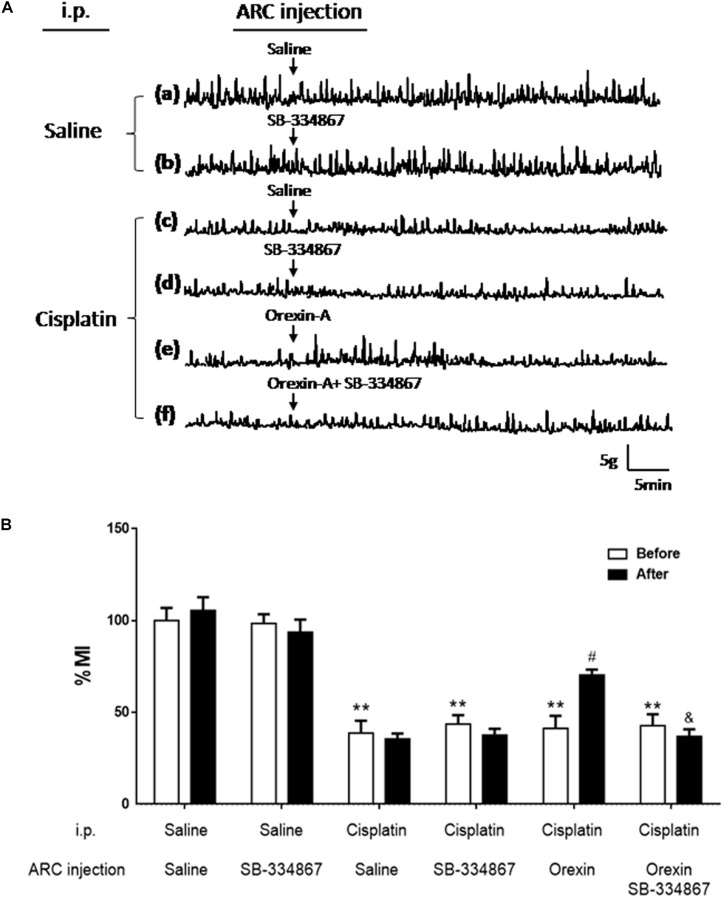
Effect of orexin-A injection into the ARC on gastric motility in cisplatin-treated rats. Orexin-A (0.5 μg) increased gastric motility that was inhibited by cisplatin in rats, and this effect was blocked by the orexin receptor 1 antagonist SB334867. **(A)** Recording curves of gastric motility: gastric motility was inhibited by cisplatin compared with SS group in rats **(a,c)**. However, the inhibitory effects induced by cisplatin were recovered, following injection of orexin-A (0.5 μg, **e**) into the ARC, and blocked by the orexin receptor 1 antagonist-SB334867 **(f)**. Administration of SB334867 alone did not significantly alter gastric motility in normal and cisplatin-treated rats **(b,d)**. **(B)** Changes in the percentage gastric motor index (% MI) of cisplatin-treated rats caused by injection of orexin into the ARC are shown. ^∗∗^*P* < 0.01 vs. SS group; ^#^*P* < 0.05 vs. CS group; ^&^*P* < 0.05 vs. CSB group, *n* = 10. ARC, arcuate nucleus; CS, cisplatin + saline; CSB, cisplatin + SB-334867; SS, saline + saline.

Figure 2Ac shows that gastric motility was significantly inhibited by cisplatin. The gastric % MI of the CS group was reduced to 34.72 ± 7.47% (*P* < 0.01 vs. the SS group, Figure 2B). In the CO group, orexin-A accelerated gastric motility (Figure 2Ae) and increased the % MI to 70.64 ± 9.17% (*P* < 0.05, vs. the CS group, Figure 2B). Administration of SB-334867 blocked the effect of orexin-A on gastric motility and the % MI of the COSB group was reduced to 39.26 ± 5.20% (*P* < 0.05 vs. the CO group, Figures 2Af,B). Administration of SB-334867 alone did not affect gastric motility (*P* > 0.05, vs. the SS group, Figures 2Aa,b,d). These findings suggest that injection of orexin-A into the ARC promoted gastric motility that was initially inhibited by cisplatin, and exerted these effects via the orexin-1 receptor.

### Unilateral PVN Lesions Block the Effects of Activation of the Ipsilateral ARC OX-1R-Expressing Neurons

**FIGURE 3:**
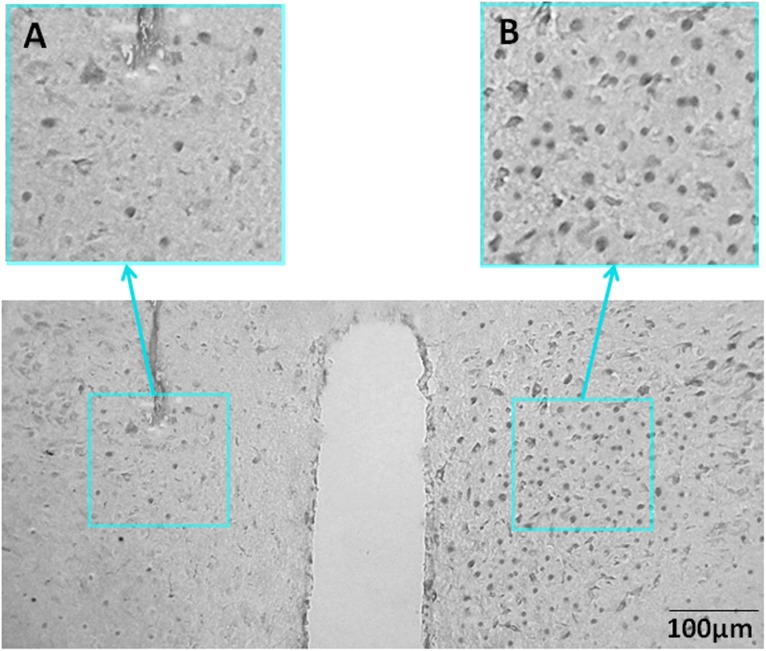
Representative unilateral PVN lesions. **(A)** NeuN immunohistochemistry staining for a representative unilateral lesion targeting the PVN. **(B)** NeuN immunohistochemistry staining contralateral to a PVN lesion (intact side). NeuN, neuronal nuclear protein; PVN, paraventricular nucleus.

To study whether PVN was involved in the effects of orexin-A injection on kaolin intake, rats with unilateral lesions in the PVN (Figure 3) were implanted with bilateral ARC-targeted cannulas.

On the third day after cisplatin injection, kaolin intake was measured following unilateral administration of orexin-A to the ARC of rats with PVN or sham lesions that were either contralateral or ipsilateral to the site of orexin-A injection.

**FIGURE 4:**
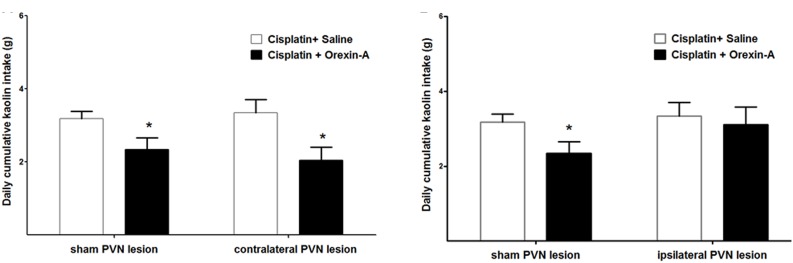
Effect of PVN unilateral disconnection on ARC orexin-A-mediated kaolin consumption in cisplatin-treated rats. Cumulative kaolin intake following unilateral ARC injection of orexin-A (0.5 μg) that was: **(A)** contralateral to a unilateral PVN lesion and a sham lesion, **(B)** ipsilateral to a unilateral PVN lesion and a sham lesion. Data are presented as mean ± SEM; ^∗^*P* < 0.05 vs. CS group, *n* = 10 in PVN lesion group and *n* = 7 in sham lesion group. ARC: arcuate nucleus; PVN: paraventricular nucleus; CS: cisplatin + saline.

Orexin-A that was injected to the ARC, contralateral to the PVN lesion (or sham lesion) markly reduced kaolin intake in both groups (2.34 ± 0.32 vs. 3.18 ± 0.21 g and 2.05 ± 0.35 vs. 3.34 ± 0.37 g, *P* < 0.05 vs. saline, Figure 4A). Whereas orexin-A injected to the ARC, ipsilateral to the PVN lesion (or sham lesion) obviously reduced kaolin intake in the sham group alone (3.45 ± 0.22 vs. 2.14 ± 0.27 g, *P* < 0.05, Figure 4B). These results show that neural communication between the ARC and the PVN is required for the effects of ARC orexin-mediated kaolin intake to be manifested.

### OX1R-Expressing ARC Neurons Project to PVN Neurons

The ARC to PVN pathway, hypothesized to take part in orexin receptor signaling for the control of kaolin intake, was investigated using FG retrograde tracing and immunohistochemistry.

**FIGURE 5:**
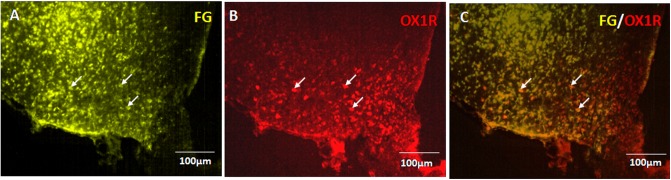
OX1R-expressing ARC neurons provide input to PVN neurons. **(A)** Retrogradely labeled Fluoro-Gold in ARC. **(B)** OX1R expression. **(C)** FG and OX1R co-labeled soma; *n* = 8. ARC, arcuate nucleus; OX1R, orexin receptor type 1.

Seven days after injection of FG into the PVN, abundant retrograde labeling was observed in the ipsilateral ARC exclusively (Figure 5A). Consistent with previous reporting extensive OX1R expression in the ARC (Rodgers et al., 2002; Martynska et al., 2005). The immunohistochemistry results confirmed OX1R protein expression in ARC neurons (Figure 5B). Retrograde labeling and immunostaining demonstrated that many FG retrogradely labeled ARC neurons also expressed OX1R (Figure 5C). The percentage of FG positive neurons that also expressed OX1R showed 27.81% colocalization in rats injected with FG in the PVN. These data indicate that part of the ARC neurons that project to the ipsilateral PVN also express OX-1R.

### Orexin-A Injection Into the ARC Increases c-Fos Expression in PVN NPY-Expressing Neurons

**FIGURE 6:**
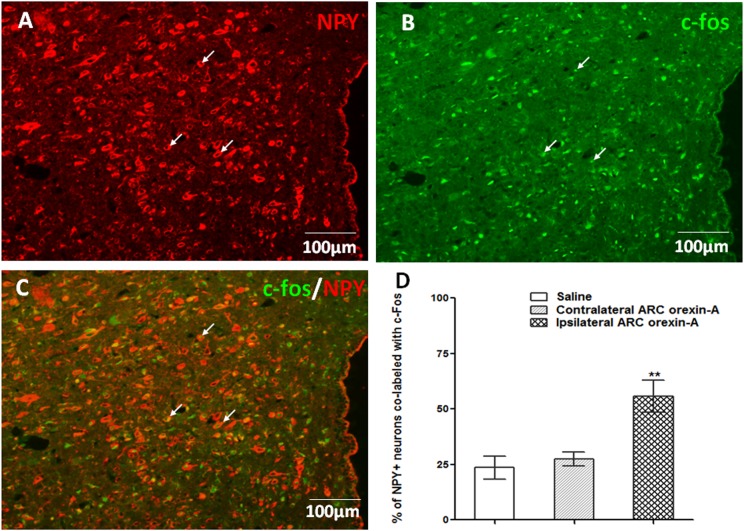
Orexin-A injection into the ARC increases c-Fos expression in PVN NPY-expressing neurons. **(A)** NPY-expressing neurons (red) in the PVN. **(B)** Expression of c-Fos (green) in the PVN induced by ipsilateral orexin-A (0.5 μg) administration into the ARC. **(C)** Co-labeling of PVN NPY expression and c-Fos expression. **(D)** Quantification of NPY positive neurons co-labeled with c-Fos, where co-labeling was significantly higher compared to saline or contralateral orexin-A injections into the ARC. Data are presented as mean ± SEM, ^∗∗^*P* < 0.01, *n* = 8. ARC, arcuate nucleus; NPY, neuropeptide Y; PVN, paraventricular nucleus.

In comparison to injections of saline or contralateral orexin-A, unilateral orexin-A injection into the ARC significantly increased c-Fos expression, in the ipsilateral PVN (*P* < 0.01 vs. saline treatment and contralateral PVN). Notably, orexin-A injection into the ARC activated about 55% of the NPY-expressing neurons in the ipsilateral PVN (*P* < 0.01 vs. saline and contralateral PVN treatment, Figure 6). These data corroborate the hypothesis that ARC orexin signaling activates NPY-expressing PVN neurons.

### Central Blockade of NPY-R Eliminates ARC Orexin-Mediated Inhibition of Kaolin Intake

**FIGURE 7:**
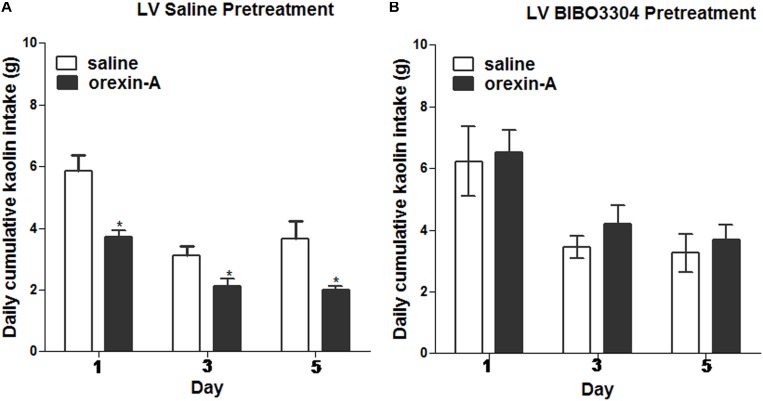
Effects of NPY-1 receptor blockade on ARC orexin-mediated inhibition of kaolin intake. Cumulative kaolin intake following: **(A)** pretreatment with saline in the lateral ventricle (LV), followed by orexin-A (0.5 μg, unilateral) administration into the ARC; or **(B)** LV NPY-1 receptor antagonist, BIBO3304 (60 μg), followed by orexin-A administration into the ARC. Data are presented as mean ± SEM; ^∗^*P* < 0.05, *n* = 10. ARC, arcuate nucleus; NPY, neuropeptide Y.

To test whether the inhibiting effects of orexin-A injection on kaolin intake induced by cisplatin requires downstream NPY signaling, a lateral ventricular injection of either saline or the NPY1 receptor antagonist, BIBO3304 (at a dosage of 60 μg) was administered to rats. Immediately thereafter, the rats received unilateral parenchymal ARC injections of orexin-A (0.5 μg, 0.5 μL) or saline into the ARC. Orexin-A significantly reduced kaolin intake in cisplatin-treated rats, pretreated with saline in the lateral ventricular, but had no effect relative to saline on kaolin intake in cisplatin-treated rats that had been pretreated with BIBO3304 (Figure 7; *P* < 0.05 vs. saline pretreatment). The above data suggest that ARC-mediated effects are associated with downstream NPY1 receptor signaling.

## Discussion

As a drug that is widely used for malignant tumors, the major side effects of cisplatin are nausea and vomiting. Serotonin receptors are reportedly associated with the vomiting caused by cisplatin, and 5-HT3 receptor antagonist steroids and metoclopramide have been used to alleviate these effects (Percie du Sert et al., 2011). Furthermore, cisplatin induces mainly the anorexinergic changes of neuropeptides. Among these peptides, orexin levels become increased in the LHA following cisplatin treatment (Yoshimura et al., 2013), showing a distinct difference in function from the other peptides.

Orexin-containing neurons are mainly distributed in the dorsal and LHAs. However, their projections and orexin receptors are widely distributed in the brain, including the ARC, PVN, septal nuclei, and thalamus (Peyron et al., 1998). As the ARC is a sensor of metabolic signals, OX1R-containing neurons can be found throughout the nucleus (Guan et al., 2002). Based on these findings, we studied the effect of exogenous orexin in the ARC on emesis caused by cisplatin. The orexin-A injection into the ARC reduced kaolin intake. Kaolin intake, typically used to evaluate cisplatin-induced emesis in rats and the anti-emetic efficacy of newly developed drugs in preclinical studies (Malik et al., 2007; Yamamoto et al., 2014), revealed that the emesis caused by cisplatin was improved by orexin-A.

Similar to the facilitating effects of orexin-A on gastric motility in normal rats (Krowicki et al., 2002; Sun et al., 2016), orexin-A improved gastric motility that was inhibited by cisplatin. Cisplatin induces the retro-propagation of gastrointestinal motor contractions from the jejunum to the stomach, accompanied by emesis (Ando et al., 2014). Based on the characteristics of the stomach, the effect of orexin-A on gastric motility could promote gastric emptying and reduce reflux. These effects are believed to comprise a mechanism to reduce kaolin intake.

To investigate the functional relationship of a putative ARC to PVN connection, we combined behavioral neuropharmacology (orexin-A injection into the ARC) with a unilateral LHA lesion-mediated neural disconnection approach. This approach applies an exclusively ipsilateral connection between the ARC and the PVN. The results showed that orexin-A injection into the ARC, ipsilateral to unilateral PVN lesions (ARC OX1R to PVN communication eliminated), blocks ARC orexin-inhibited kaolin intake that occurs in animals of sham lesions group, and also happens when orexin-A administrations into the ARC are contralateral to the unilateral PVN lesion (ARC OX1R to PVN communication intact). These results suggest that ARC to PVN communication is important for the inhibitory effect of orexin injection into the ARC on kaolin intake.

Furthermore, FG retrograde tracing and immunohistochemistry experiments were used to determine whether there are neural fibers that conduct signals between the ARC and PVN. The colocalization of FG and OX1R in the ARC and the increased c-Fos expression in the PVN induced by ARC stimulation suggest that ARC neurons are directly involved in orexinergic signaling to the PVN. Our neuroanatomical data are consistent with those of previous studies that show that metabolic signals are sensed by interoceptive neurons of the ARC and ventromedial nuclei of the hypothalamus that send synaptic projections to the PVN. As a major hypothalamic nucleus of autonomic regulation, the PVN is the site at which metabolic signals are integrated to control feeding behavior, via projections to the rostral ventrolateral medulla and spinal cord (Dampney et al., 2002; Pyner, 2009; Cassaglia et al., 2014; Zaman et al., 2014).

We examined the requirements of downstream NPY signaling for the ARC orexin-mediated effects on kaolin intake. We demonstrated that central NPY receptor blockade (via lateral ventricular injections of the NPY receptor antagonist) decreased the effects of ARC orexin-A signaling. However, because ventricular injections spread throughout the central nervous system, and NPY receptors distribute throughout the brain (Dumont et al., 1998; Caberlotto et al., 2001), we can only suppose about the mediating sites of NPY receptor signaling.

Previous study has proved that blockage of NPY receptors in the ARC decreases food intake induced by lateral ventricular orexin-A injection (Yamanaka et al., 2000). Based on these findings and the effects of NPY signaling in various fields of feeding behavior (Higuchi, 2012; Kohno and Yada, 2012; Loh et al., 2015), we presume that PVN NPY signaling is an important downstream target of ARC orexin. In addition to providing an input to the PVN, ARC neurons also project to some other feeding-related brain nucleus, such as the LHA, supraoptic nucleus, preoptic nucleus, and suprachiasmatic nucleus (Elias et al., 1999; Saeb-Parsy et al., 2000; Douglas et al., 2002; Sanathara et al., 2014). These nucleus also have a topographically organized output to the PVN, and neural connection between the LHA-PVN and supraoptic nucleus-PVN has been found to be essential for feeding and gastric motility (Silverman et al., 1981; Uschakov et al., 2006; Garcia-Luna et al., 2010; Sanathara et al., 2014). Further studies are required to determine the functional relevance of the ARC and other nuclei and the possible pathways associated with orexin and appetite.

**FIGURE 8:**
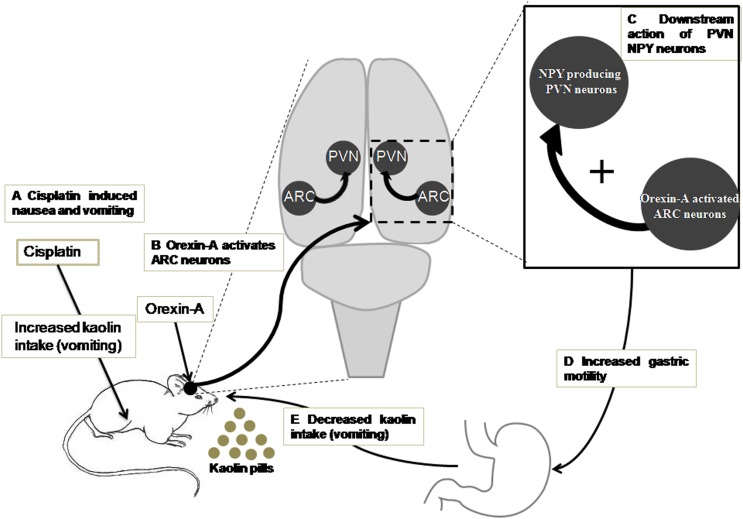
A model for ARC orexin-mediated inhibition of kaolin intake in cisplatin-treated rats. **(A)** Kaolin intake, an indicator of vomiting, was increased by intraperitoneal injection of cisplatin. **(B)** Orexin-A acted on OX1R in ARC neurons that engaged **(C)** downstream activation of PVN NPY neurons and CNS NPY-1R signaling. **(D,E)** Activation of this neural pathway increased gastric motility and reduced kaolin intake induced by cisplatin. ARC, arcuate nucleus; NPY, neuropeptide Y; OX1R, orexin receptor type 1; PVN, paraventricular nucleus.

According to the present data and the studies of previous related research, we propose the following sketch map, in which ARC orexin signaling regulates the inhibitory effects of cisplatin on kaolin intake (Figure 8): (i) orexin-A injection into the ARC reduced kaolin intake induced by cisplatin; (ii) orexin-A injection into the ARC increased gastric motility inhibited by cisplatin; (iii) ARC OX1R-expressing neurons project to the PVN; (iv) orexin-activated neurons in the ARC engage downstream PVN NPY-expressing neurons; and (v) activation of this neural pathway reduces kaolin intake induced by cisplatin. This study highlights novel neurobiological mechanisms, by which orexin is involved in ARC-PVN circuitry in the brain that in turn, modulates hypothalamic systems and gastric motility to alleviate the emesis induced by cisplatin.

## Author Contributions

LX, SG, and FG were responsible for the conception, design, writing, and revision of the article. FG, SG, XS, YG, and XL acquired the data. NZ undertook the statistical analysis and interpretation of data. All authors contributed to and have approved the final manuscript.

## Conflict of Interest Statement

The authors declare that the research was conducted in the absence of any commercial or financial relationships that could be construed as a potential conflict of interest.

## Abbreviations

ARC: arcuate nucleus
COSB: cisplatin + orexin-A + SB-334867
CS: cisplatin + saline
CSB: cisplatin + SB-334867
FG: Fluoro-Gold
LHA: lateral hypothalamic area
MI: motor index
NeuN: neuronal nuclear protein
NPY: neuropeptide Y
OX1R: orexin receptor type 1
PVN: paraventricular nucleus
SS: saline + saline
SSB: saline + SB-334867
